# Dental anomalies and orthodontic characteristics in patients with pseudohypoparathyroidism

**DOI:** 10.1186/s12903-019-0978-z

**Published:** 2019-12-31

**Authors:** Jane Hejlesen, Line Underbjerg, Hans Gjørup, Tanja Sikjaer, Lars Rejnmark, Dorte Haubek

**Affiliations:** 1 Section for Pediatric Dentistry, Department of Dentistry and Oral Health, Health, Aarhus University, Aarhus C, Denmark; 2 Department of Endocrinology and Internal Medicine, Aarhus University Hospital, Aarhus, Denmark; 3 Center for Oral Health in Rare Diseases, Department of Maxillofacial Surgery, Aarhus University Hospital, Aarhus, Denmark

**Keywords:** Pseudohypoparathyroidism, Dental anomalies, Enamel hypoplasia, Short root, Pulp calcification, Blunt root

## Abstract

**Background:**

Pseudohypoparathyroidism (PHP) is a rare and inherited disease caused by mutations in the *GNAS*-gene or upstream of the GNAS complex locus. It is characterized by end-organ resistance to PTH, resulting in hypocalcemia and hyperphosphatemia. We aimed to investigate the dental anomalies according to tooth types and the orthodontic characteristics of patients with PHP.

**Methods:**

Using a cross-sectional design, 29 patients (23 females) with PHP, living in Denmark, were included, and their clinical intraoral photos and radiographs were examined.

**Results:**

Pulp calcification was found in 76% of the patients. Blunting of root apex was present in 55% and shortening of root in 48% of the examined patients. Blunting and shortening of roots were seen more often in premolars than in other tooth types (p_both_ < 0.01). Crowding of lower anterior teeth was frequently observed (36%) as well as diastema in the upper arch (25%), midline diastema (18%), and Class III malocclusion (11%).

**Conclusion:**

In the present study population, the teeth were frequently affected by pulp calcification and/or deviation of the root morphology. Blunting and shortening of root(s) were more often seen in premolars than in other tooth types. Class III malocclusion was relatively prevalent. It is important to pay attention to dental anomalies and occlusion in order to provide adequate care for patients with PHP.

## Background

Pseudohypoparathyroidism (PHP) is a rare disease, characterized by hypocalcaemia with a high level of parathyroid hormone (PTH) due to PTH resistance in the target organs. The prevalence of PHP in Denmark is 1.1/100000 inhabitants [1]. PHP is caused by mutations in either *GNAS, STX16* or *GNASAS1.* All genes causing PHP are located on the maternal allele of chromosome 20q13. PHP is subdivided into different types, depending on the clinical and hormonal phenotypes: PHP type 1a (OMIM #103580), PHP type 1b (OMIM #603233), PHP type 1c (OMIM #612462), and PHP type 2 (OMIM #203330). PHP 1a, also known as Albright Hereditary Osteodystrophy (AHO), occurs due to mutations in *GNAS*. PHP 1b is caused by mutations affecting *GNAS* imprinting. A clinical phenotype of AHO without biochemical abnormalities is known as pseudopseudohypoparathyroidism (PPHP). PPHP occurs due to a mutation in *GNAS,* located at the paternal allele of chromosome 20q13. In a recent consensus report, it was recommended that an oral examination should be included regularly as part of the follow-up of patients with PHP [2].

According to a recent systematic review by our research group, the existing information on dental anomalies of patients with PHP was limited [3]. However, a number of patients with PHP have enamel hypoplasia, deviation of root morphology, and disturbance of tooth eruption. These findings were solely based on case reports and minor case series. A recent Brazilian study, investigating 19 patients with PHP [4] reported short, blunted roots in 12 (63%) patients, widened pulp chambers and/or calcified intrapulpal deposits in 4 (21%) patients, and enamel hypoplasia in 12 out of 15 (80%) patients.

In the present population-based cross-sectional study, we aimed to analyze the distribution of dental anomalies according to various tooth types and to assess the orthodontic characteristics in patients with PHP.

## Methods

### Study population

The present study was conducted from September 2014 to April 2017. The study was performed in accordance with the Declaration of Helsinki II, and all the patients gave written and verbal consent. The study was approved by Central Region of Denmark (Protocol no. 1–16–02-101-11), Danish Data Protection Agency (Protocol no. 2011-41-5955), and the Central Denmark Region Committee on Biomedical Research (Journal no. M-20110074). The study was registered at www.clinicaltrials.gov (NCT02551120).

We included 29 adults (≥18 yrs) with PHP who had been identified in a Danish epidemiological study [5] along with newly referred patients to Department of Endocrinology and Internal Medicine, Aarhus University Hospital, Denmark [1, 6]. PHP was defined as hypocalcemia with elevated PTH levels, which was not attributed to other known causes of secondary hypoparathyroidism. To ensure the medical diagnosis, medical charts from all included patients were reviewed. Among the 29 included patients (23 females, 79%), 25 had PHP and four had PPHP. They all underwent clinical oral photography and dental radiographic examination. Median age was 36 yrs. (range 21–76 yrs) (Table 1). The biochemical findings have previously been described in details [1].

**Table 1:** General characteristics of 29 patients with pseudohypoparathyroidism (PHP)

Within age category at time for the examination	Gender	Genetically-verified disease	PHP type
30’s	Female	yes	PHP1b
20’s	Female	yes	PHP1b
20’s	Male	no	PHP1b
20’s	Female	no	PHP1b
20’s	Female	no	PHP1b
30’s	Female	no	PHP1b
50’s	Female	no	PHP1b
50’s	Female	no	PHP1b
70’s	Female	no	PHP1b
20’s	Female	no	PHP1b
50’s	Female	no	PHP1b
20’s	Male	no	PHP1b
40’s	Female	no	PHP1b
40’s	Male	nd	PHP1b
30’s	Female	no	PHP1a
30’s	Female	no	PHP1a
20’s	Male	no	PHP1a
20’s	Female	yes	PHP1a
30’s	Male	yes	PHP1a
30’s	Male	yes	PHP1a
30’s	Female	no	PHP1a
20’s	Female	yes	PHP1a
30’s	Female	no	PHP1a
30’s	Female	no	PHP1a
20’s	Female	yes	PHP1a
40’s	Female	yes	PPHP
50’s	Female	yes	PPHP
20’s	Female	yes	PPHP
40’s	Female	no	PPHP

*nd* not determined/unknown

### Data collection

Clinical photos were obtained by a professional photographer (six pictures of the face, three intraoral photos of the dental occlusion, and two intraoral photos of the occlusal aspect of the upper and lower teeth, respectively). The radiographs of teeth (periapical x-rays of all teeth and panoramic radiographs) were obtained by the staff at Section of Radiology, Department of Dentistry and Oral Health, Aarhus University, Denmark. A clinical oral examination was not a part of the study protocol.

The assessment of the study material comprised a recording of dental anomalies and orthodontic characteristics. In addition, eruptional disturbances and dental treatment (e.g., fillings, crowns, and root canal treatment) were recorded. Table 2 provides the guidelines for the assessment of the dental anomalies and orthodontic characteristics [7–17]. Enamel hypoplasia was assessed according to the FDI guideline [9].

**Table 2:** Definition on dental anomalies and characteristics

Term	Definition
Deviations in the enamel formation	
Hypoplasia	Quantitative macroscopic defect of the enamel, reduced thickness of enamel. The borders of the defect should be rounded and smooth [9, 10]. The pits should be of significant size or appear more than once on the surface of the tooth.
Invagination	A clear outline of enamel inside the second maxillary incisors [16] (4.24)^a^.
Alteration of the root anatomy	
Short root	Short root is when the root appears distinctly shortened compared to mean root length [16] (4.29)^a^.
Blunting of root apex	Root ends with a clear blunting of apex [7].
Obliteration of pulp canal	The pulp canal is not visible on radiographs, because of deposits occluding the root canal [16] (4.47)^a^.
Pulp calcification	Foci of calcification in the dental pulp. Radiographically visible opaque structures in the pulp chambers. They may occur as a single dense mass or as several small radioopacities [8]. Pulp calcification was assessed on molars only.
Root flexion	A minimum of a 45 degrees bend between the axis for the apical respective the coronal part of the root.
Disturbances in the eruption or tooth number	
Impaction	Absence of tooth eruption due to an obstacle in the eruption path or ectopic position of the tooth germ [11].
Primary retention	Absence of tooth eruption without an obstacle in the eruption path or ectopic position of the tooth germ before gingival emergence [11].
Secondary retention	Arrested eruption after gingival emergence [11].
Hypodontia	Congenital absence of at least one permanent tooth or tooth germ, seen as persistence of primary teeth [15].
Hyperdontia	Teeth present in addition to the normal tooth set, seen in the permanent dentition [14].
Dental occlusion	
Sagittal molar occlusion	(if first molar is missing the canine and premolar relationship are the guide [15])
Class I	The mesiobuccal cusp of the upper first molars occludes in the mesiobuccal fossa of lower first molar [12].
Class II	The mesiobuccal cusp of the upper first molar occludes ≥ ½ width mesial to the mesiobuccal sulcus of lower first molar [12].
Class III	The mesiobuccally cusp of the upper first molar occludes ≥ ½ premolar width distal to the mesiobuccal sulcus of lower first molar [12].
Lateral cross bite	The buccal cusp of the maxillary tooth occludes lingually to the buccal cusp of the mandibular tooth; minimum two teeth in one side (M, P, C) [16] (4.62)^a^.
Open bite	Vertical distance between incisal edges of incisors perpendicular to occlusal plane > 0 [46] (4.61)^a^.
Overbite, increased	Maxillary anterior teeth cover the crown of the mandibular teeth totally [16] (4.63)^a^.
Crowding of teeth	Deficit of space in the dental arch visible by severely rotated teeth and/or buccally or lingually displaced teeth [16] (4.57)^a^
Midline diastema	Space between the upper central incisors > 1 mm.
Spaced teeth	Diastema in multiple places (≥4) in the lower or the upper dental arch [16] (4.58)^a^.
Ectopic position	Tooth totally displaced outside the normal position in the dental arch [13].

^a^Figures in parentheses are the respective paragraphs in La Dure-Molla 2019 [16]

Three of the authors (JH, HG, and DH) were calibrated and involved in the assessment of the dental and orthodontic characteristics (i.e., a complete analysis and evaluation of the intraoral photos of nine patients (31%)). Third molars were excluded from the data collection. An additional file provides data for the initial calibration (see Additional file 1). After the initial calibration, the guidelines were discussed and revised, i.e., the root flexion had to be more than 45 degrees to be included. The revised guideline for definitions on dental anomalies and characteristics are provided in Table 2.

Beyond the nine patients assessed for the calibration, JH performed the analysis and evaluation of the additional 20 patients. In case of doubt, consensus was reached among the investigators.

### Statistical methods

Each of the dental anomalies was recorded as present or absent at tooth- and subject level. For each type of dental anomaly, the relative number of affected teeth according to the total number of teeth present in the mouth was calculated and used for further analyses. Dental occlusion and other orthodontic characteristics were assessed at subject level.

The presence of dental anomalies, root shortening, and/or root blunting according to tooth types (I, C, P and/or M), were compared by chi square tests. Results are reported as the number of affected patients and/or teeth with percentages (%) of the respective total numbers. *P*-values < 0.05 were considered statistically significant. All analyses were performed using Stata/IC 13.1® (StataCorp LP 2013. College Station, TX 77845, USA) and Microsoft® Excel® 2016.

## Results

Dental radiographs were obtained for all 29 patients, and clinical photos were obtained in 28 patients. Among the 29 patients, the number of teeth present in the oral cavity varied between 20 and 31 (mean 26.6; SD 2.5). In total 767 (excluding third molars, one primary tooth, and three supernumerary teeth) were assessable on radiographs. According to tooth types, 231 incisors, 113 canines, 210 premolars, and 213 M were assessed. The total number of teeth assessable on clinical photos was 741. The dental anomalies present are shown in Table 3 and the orthodontic characteristics in Table 4. For comparison, Table 4 includes reference values, originated from a Danish epidemiological study by Helm [17].

**Table 3:** Dental characteristics in 29 patients with pseudohypoparathyroidism (PHP)

Dental	Patients (%)	Teeth (%)
	*n* = 29	*n* = 767
Enamel hypoplasia	8 (29)^a^	14 (2)^a^
Shortening of root	14 (48)	87 (11)
Blunting of root apex	16 (55)	78 (10)
Root flexion	12 (41)	14 (2)
Pulp calcification in molar	22 (76)	115 (54)^b^
Obliterated pulp canal	3 (10)	4 (1)
Tooth crown size/shape		
Microdontia	2 (7)	3 (0)
Macrodontia	1 (3)	1 (0)
Peg-shaped	2 (7)^a^	3 (0)^a^
Screwdriver-shaped	3 (11)^a^	3 (0)^a^
Tuberculum Carabelli	3 (11)^a^	7 (1)^a^
Radix relicta	1 (3)	1 (0)
Primary retention	2 (7)	2 (0)

Figures are numbers (n) of patients respective teeth followed by percentages of the total number (%)

^a^Oral clinical photos of 28 patients with a total of 741 teeth

^b^Recorded for molars, only (*n* = 213)

**Table 4:** Dental occlusion and crowding/diastema in 28 patients with pseudohypoparathyroidism (PHP)

	Patients with PHP (%) *n* = 28		Reference material^a^ (%) (Male-Female)
Midline diastema	5 (18)		–
Diastema upper	7 (25)		8.7–4.6
Diastema lower	4 (14)		5.5–2.7
Crowding lower anterior	10 (36)		31.0–30.7
Crowding upper anterior	3 (10)		19.4–25.5
Deep bite	2 (7)		22.7–14.5
Crossbite			9.4–14.1
Unilateral crossbite	1 (3)		–
Bilateral crossbite	2 (7)		–
Molar occlusion	Right^b^	Left	*–*
Class I	20 (71)	21 (75)	–
Class II	5 (18)	4 (14)	25.2–25.8
Class III	2 (7)	3 (11)	4.1–4.5

Number of patients (n) followed by percentages within group (%)

^a^Prevalence of the trait in a population of 1240 (565 males and 675 females, respectively) [17]

^b^Missing assessment in one patient due to the absence of multiple teeth in the right side

None of the patients had molar incisor hypomineralization [18], fusion of teeth, invagination, single median maxillary central incisor, or ectopic position of teeth. Furthermore, the following characteristics occurred in single patients only and were, therefore, not included in Tables 3 and 4: a persistent primary tooth, dental impaction, hypodontia, hyperdontia, widened pulp canal, hypercementosis, a cyst, crowding in other places of the dental arch than in the anterior segment, and anterior open bite.

**Fig. 1:**
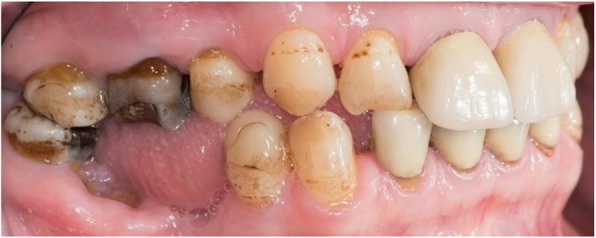
Enamel hypoplasia (17, 16, 13, 12, 43), amalgam fillings (16, 47), composite filling (44), and porcelains crowns (11, 21, 32, 31, 41, 42) in a 58-year old female

Table 3 shows the dental characteristics of the study population. The most prevalent dental anomalies in the study group were pulp calcification (76%), blunting of the root apex (55%), and shortening of the root (48%). Figure 1 shows an example of the root anomalies found.

**Fig. 2:**
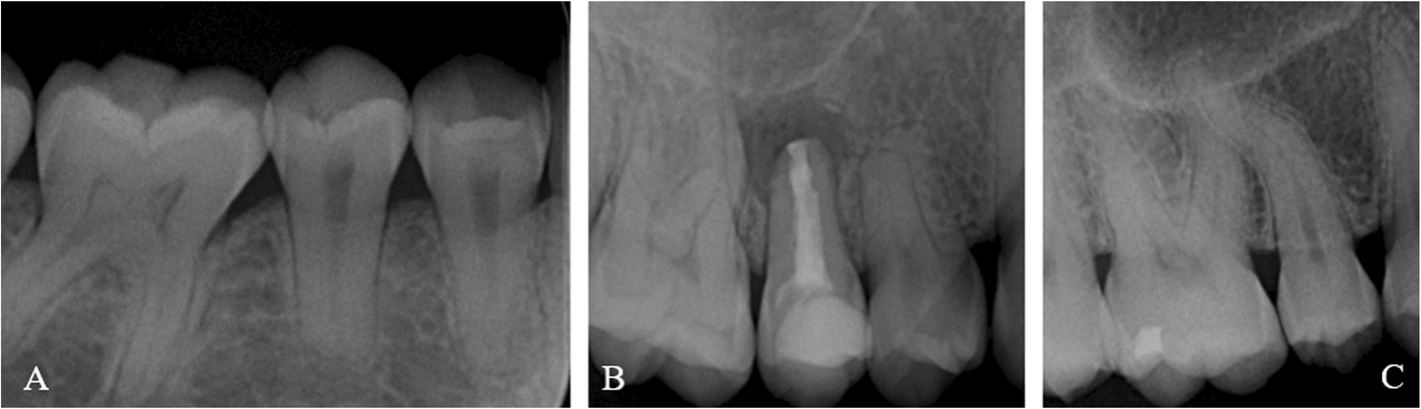
**a** and **b** Blunt root and short root (14, 15, 44, 45), pulp calcification (16, 46), root canal treatment, and apical periodontitis (15) in a 21-year old man. **c** Root flexion (15) and pulp calcification (16) in a 45-year old woman

The prevalence of teeth with enamel hypoplasia (Fig. 2), short roots and/or blunt apices according to different tooth types (I, C, P, M) are shown in Table 5. A significant difference was found in the distribution of short roots and blunting of root apices, which occurred most often in premolars (*p* < 0.01).

**Table 5:** The distribution of enamel hypoplasia, blunt root(s), shortening of root(s) according to tooth type in 767^a^ teeth in 29 patients with pseudohypoparathyroidism (PHP)

Type of tooth disturbance	Incisors n (%)	Canines n (%)	Premolars n (%)	Molars n (%)	Total n (%)	*p*-value^b^
Total	231	113	210	213	767	
Enamel hypoplasia	5 (2.2)	2 (1.8)	2 (1.0)	5 (2.3)	14 (1.8)	0.719
No enamel hypoplasia	212 (91.8)	107 (94.7)	196 (93.3)	197 (92.5)	712 (92.8)	0.994
NA	14 (6.1)	4 (3.5)	12 (5.7)	11 (5.2)	41 (5.3)	0.807
Blunt root	8 (3.5)	7 (6.2)	57 (27.1)	6 (2.8)	78 (10.2)	< 0.001
No blunt root	223 (96.5)	106 (93.8)	153 (72.9)	207 (97.2)	689 (89.8)	0.024
NA	0 (0.0)	0 (0.0)	0 (0.0)	0 (0.0)	0 (0.0)	
Shortening of root	10 (4.3)	8 (8.0)	58 (27.6)	11 (5.2)	88 (11.5)	< 0.001
No shortening of root	221 (95.7)	105 (92.9)	152 (72.3)	202 (94.8)	679 (88.5)	0.033
NA	0 (0.0)	0 (0.0)	0 (0.0)	0 (0.0)	0 (0.0)	

*NA* Not possible to assess demarcated opacities/hypoplasia/blunt root/shortening of root (see text)

^a^The number of permanent teeth present in patients varied from 21 to 28 (excluding supernumerary teeth and third molars)

^b^The expected value (n) for the respective tooth groups, if the specific characteristic was distributed equally according to the number of teeth in each of the tooth groups, was tested

Six incisors, five premolars, and 1 molar had an artificial crown, 1 molar had an extensive filling, 1 molar had lost its crown, and 1 molar was retained in the jaw. Thus, the characteristics of the natural tooth crowns of these 14 teeth were not assessable. One patient did not have oral photos taken, as she did not show up for her appointment. Consequently, the crown of eight incisors, four canines, seven premolars, and seven molars present in that patient were not assessed on clinical photos. None of the patients with PHP had fixed or partial dentures, but one patient had a dental implant inserted.

The most prevalent orthodontic traits in the patients with PHP were crowding of the lower anterior teeth (36%), diastema in the upper arch (25%), midline diastema (18%), and Class III molar occlusion (11%). However, the most prevalent molar occlusion was normal Class I (right side: 71% and left side: 75%).

## Discussion

To our knowledge, the present study represents the largest and the first cross-sectional study with a systematic analysis of the dental anomalies and orthodontic characteristics of patients with PHP. In our group of patients with PHP, the most prevalent types of dental anomalies were pulp calcification, blunting of root apex, and shortening of root.

The majority of former dental studies on patients with PHP are case reports or minor case series. In a recently published systematic review on dental anomalies in PHP [3], enamel hypoplasia occurred in 73% of the reported cases. In contrast, we found only eight (29%) patients with enamel hypoplasia. None of the studies included in the systematic review [3] described the diagnostic criteria used. Therefore, it is uncertain if the authors used the Developmental Defect of Enamel index (DDE criteria) defined by the Fédération Dentaire Internationale, FDI group [9], or some other classification system. Furthermore, decades ago the terminology ‘internal and external enamel hypoplasia’ was commonly used [19]. Such changes in the definition of dental anomalies may have led to the diagnosis ‘enamel hypoplasia’ of lesions that we today would have diagnosed as ‘enamel opacities’. Therefore, due to the changes over time of the definitions of dental anomalies, the comparison of our results with results of previous studies has to be done with caution.

In the present study, we found a higher frequency of pulp calcification, blunting of root apex, and shortening of root than previously reported [3]. However, only one of the previous studies included more than six patients [4]. The study by Reis and coworkers [4] showed shortening and blunting of root apices at a similar level as in the present study. Thus, it appears that root anomalies are relatively prevalent in patients with PHP. The findings in the present study were not adjusted for age. The exact influence of age is beyond the scope of the authors’ current methodology, but would be of interest for further multi-center studies.

Calcium and phosphate homeostasis are considered to be of significance in the development of teeth, and hypocalcemia is hypothesized to be involved in the etiology of enamel hypoplasia [20]. Teeth develop in a predicable way over 20 years, and the formation of teeth can be used to estimate age up to early adulthood [21]. Therefore, disturbances of the biological processes related to tooth development can cause defects of the tooth crown according to the particular time point of the insult. Previous studies have shown that enamel defects are present in children who suffered from hypocalcemia during the period of enamel formation [22–25]. Fraser and Nikiforuk [26] support this finding. However, they found no correlation between plasma phosphate concentration and enamel defects [26]. In an animal study [27], enamel defects developed in rats with induced hypocalcemia. Although a previous animal study had shown hypocalcemia’s effect on enamel, the likely connection between enamel defects and age of PHP diagnosis and initiating medical treatment in humans, has not been established so far [4]. The PTH resistance is often absent at birth so the resistance to PTH and hypocalcemia often develops over time [28]. A connection between types of PHP and the time point for development of PTH resistance and hypocalcemia has not been established [28]. However, normal tooth formation may not only depend on calcium metabolism. In mice, *GNAS* and *STX16* are highly expressed during tooth germ formation [29]. Therefore, a direct effect of *GNAS* and *STX16* on tooth development in humans is possible. More research on that topic is needed to obtain a better understanding of the mechanism(s) behind defects in the enamel formation during tooth formation.

In the present study, short root and blunting of root apices were most prevalent in premolars. The root anomalies were less prevalent in second molars compared to premolars, although they develop during the same time period [30]. According to Kjaer [31], the theories of different developmental fields of the jaw may help to explain this. Each of the developmental fields has a common embryologic origin, and each of them contains specified tooth types. Cells of the different parts of the neural crest migrate together with the innervation to different parts of the cranium and dentition. The dental anomalies in the maxillary respective palatine developmental field might indicate developmental disturbances associated with the differentiated innervation of these fields [31]. The maxillary field includes the premolars and the canines, and the presence of root anomalies is not significantly different between incisors, canines and molars. However, there is a tendency for canines to have blunting of apices (*p* = 0.07) and short roots (*p* = 0.11) more often than incisors and molars. Therefore, it cannot be excluded that the distribution of dental anomalies in patients with PHP might be affected by unspecified differences in the development of the palatine field respective to the maxillary field.

According to our assessment of dental occlusion, the incisal relationship does not seem to be affected by PHP, as only one patient showed open bite, and only two patients showed deep bite. In a previous Danish epidemiologic study on adolescents [17], it was stated that 2.3% of males and 1.8% of females had an anterior open bite. This is similar to our findings in the PHP group. However, deep bite is much more common in the reference material (14.5–22.7%) [17] than in the patients included in the present study (7%) (Table 4). The majority of patients with PHP in the present study showed normal sagittal occlusion. Out of the patients, who deviated from the normal Class I occlusion a relatively large proportion showed Class III occlusion (7–11%), although it did not reach the level of Class II occlusion (14–18%). This is in contrast to a normal Caucasian population, in which Class II occlusion (25–26%) is much more prevalent than Class III malocclusion (4–5%) [17]. However, in the present study, the number of patients with deviation from normal Class I occlusion was very small, and deviations from the reference material might be incidental. Thus, Class III malocclusion cannot be highlighted as a common trait in patients with PHP.

According to the present study, crowding of teeth in the anterior section of the lower dental arch did not appear to be prevalent in PHP, as the prevalence of this phenomenon was at a similar level compared to the reference material [17]. In contrast, crowding of teeth in the anterior section of the upper dental arch was less prevalent in the study population compared to the reference material [17]. Diastema in both the upper and lower dental arches seemed to be more prevalent in patients with PHP compared to the reference material. In the present study, 25% of patients with PHP had upper diastema compared to only 8.7 and 4.6% for males respective females in the reference material [17]. Therefore, spacing of the dentition might be a trait for patients with PHP.

It is important for dentists to be aware of the dental anomalies associated with PHP. Concerning dental caries, several potential risk factors could be suggested. For example, enamel defects, such as hypoplasia, can lead to disintegrated enamel [10]. This may retain dental plaque and thereby increase the risk of dental caries. An association between presence of enamel defects and dental caries has previously been stated in the literature [32, 33]. It is also likely that oral hygiene procedures, especially in children, are overlooked because of the concomitantly occurring medical condition, which calls for full attention by the child and parents [34]. This situation may eventually lead to food remnants and plaque accumulation. In the present study, the presence of dental plaque and caries was not assessed. Thus, the impact of such factors cannot be evaluated in the present study.

According to dental treatment, it is important to consider the presence of short roots before initiation of orthodontic treatment, as any orthodontic treatment includes the risk of inducing root resorption [35]. Expectably, short roots are particularly vulnerable to orthodontically-induced root resorption because of the presence of the short root already from the initiation of the orthodontic treatment.

A major strength of our study is that the dental findings have been evaluated both on clinical oral photos and on dental radiographs. The combination of different techniques used gives a more precise characterization of the dental status. In the present study, the root morphology was assessed on two-dimensional (2D) radiographs. The usage of uni-directional pictures in the interpretation of root morphology is, however, a limitation. To minimize the irradiation risk, we decided not to obtain three-dimensional (3D) radiographs of the teeth by cone beam computer tomography (CBCT). However, CBCT would have left us with more exact and detailed information on the root morphology. Therefore, we cannot fully exclude that more than 14 teeth had a root bending at or above 45 degrees (Table 3).

Another limitation of our study was the diverse nature of the patients studied. Not all patients had their PHP diagnosis genetically verified. Despite the population-based data collection and the study population being relatively large, the sample size was still limited (*n* = 29) due to the rarity of the disease. Therefore, we were not able to perform a comparison between the different subtypes of patients due to the increased risk of type II errors. Furthermore, the lack of a control group was a limitation to the study, we have, however, compared the findings of orthodontic characteristics to norm-based material. Unfortunately, similar norm-based material does not exist concerning dental anomalies, although single entities have been reported on in a number of different control groups and study populations [36–39]. Dental anomalies as agenesis and delayed eruption were not possible to diagnose in the present cross-sectional study on adults, as dental history of the patients were not known and we only included adults. Therefore, agenesis of a permanent tooth was diagnosed only when the corresponding primary tooth persisted. Delayed eruption has been reported in previous studies [40–46]. As we examined adults only and due to their dental history being unknown, the assessment of delayed eruption was not a part of the present study. To investigate eruption disturbances and dental agenesis, a study on children with PHP is required.

## Conclusion

The teeth in the present study population were frequently affected by pulp calcification and/or deviation of the root morphology. Blunting and shortening of root were more often seen in premolars than in other tooth types. Class III occlusion was relatively prevalent. It is important to pay attention to dental anomalies and occlusion in order to provide adequate care for patients with PHP.

## Supplementary information

**Additional file 1: Table S1.** Initial calibration of the three examiners: assessment of dental and orthodontic characteristics of randomly chosen nine patients with PHP

## Data Availability

The data that provide the basis for the present study is available by contact to the corresponding author. Restrictions apply to the availability of these data and to a certain time period, as the data were used under license for the current study, and so are not publicly available.

## Abbreviations

2D: Two-dimensional
3D: Three-dimensional
AHO: Albright Hereditary Osteodystrophy
C: Canines
I: Incisor
M: Molar
P: Premolar
PHP: Pseudohypoparathyroidism
PPHP: Pseudopseudohypoparathyroidism
PTH: Parathyroid hormone
SD: Standard deviation
