# Supplementary material for: Dental anomalies and orthodontic characteristics in patients with pseudohypoparathyroidism
Source: BMC Oral Health. 2019 Dec 31;20:2. doi: 10.1186/s12903-019-0978-z (PMC6938634; doi:10.1186/s12903-019-0978-z)
Supplement: Supplementary file 1 — Additional file 1: Table S1. Initial calibration of the three examiners: assessment of dental and orthodontic characteristics of randomly chosen nine patients with PHP [file 12903_2019_978_MOESM1_ESM.docx]

**Additional material**

| **Additional file 1: Table S1.** | |  |  |  |  |  |  |  |  |  |  |  |  |
| --- | --- | --- | --- | --- | --- | --- | --- | --- | --- | --- | --- | --- | --- |
| Initial calibration of the three examiners: assessment of dental and orthodontic characteristics of randomly chosen nine patients with PHP | | | | | | | | | | | | | |
| Patient id | | | 1 | 2 | 3 | 4 | 5 | 6 | 7 | 8 | 9 | Agreement*  n/total (%) | |
|  | | |  |  |  |  |  |  |  |  |  |  |  |
| Dental characteristics | | |  |  |  |  |  |  |  |  |  |  |  |
| Enamel hypoplasia | | | 0,0,0 | 0,0,0 | 0,0,0 | 0,0,0 | 0,0,0 | 0,0,0 | 0,0,0 | 3,3,3 | 0,0,0 | 236/248 (95.2) |  |
| Invagination | | | 1,0,0 | 1,0,0 | 0,0,0 | 0,0,0 | 0,0,0 | 0,0,0 | 0,0,0 | 0,0,0 | 0,0,0 | 246/248 (99.2) |  |
| Shortening of root | | | 0,0,0 | 0,0,0 | 0,0,0 | 0,0,0 | 1,1,3 | 5,3,3 | 0,1,0 | 2,0,0 | 0,3,0 | 238/248 (96.0) |  |
| Blunting of root apex | | | 0,0,0 | 0,0,0 | 0,0,0 | 0,0,0 | 2,1,0 | 5,4,0 | 0,0,0 | 0,2,2 | 0,2,0 | 237/248 (95.6) |  |
| Root flexion | | | 2,1,0 | 8,5,2 | 5,4,0 | 6,3,0 | 4,1,0 | 3,4,0 | 12,9,0 | 4,6,6 | 4,9,1 | 199/248 (80.2) |  |
| Pulp calcification in molar | | | 8,6,8 | 7,6,8 | 0,1,0 | 1,3,5 | 3,2,3 | 2,1,0 | 7,7,3 | 0,0,0 | 6,8,4 | 228/248 (91.9) |  |
| Obliterated pulp canal | | | 0,0,0 | 0,0,0 | 0,0,0 | 0,0,0 | 1,0,0 | 0,0,0 | 0,0,0 | 0,0,0 | 0,0,0 | 247/248 (99.6) |  |
| Tooth crown size/shape | | |  |  |  |  |  |  |  |  |  |  |  |
|  | Microdontia | | 0,0,0 | 0,0,0 | 0,0,0 | 0,0,0 | 1,1,0 | 0,0,0 | 0,0,0 | 0,0,0 | 0,1,0 | 246/248 (99.2) |  |
|  | Macrodontia | | 0,0,0 | 0,0,0 | 0,0,0 | 0,0,0 | 0,0,0 | 0,0,0 | 0,0,0 | 0,0,0 | 1,0,0 | 247/248 (99.6) |  |
| Peg-shaped | | | 0,0,0 | 0,0,0 | 0,0,0 | 0,0,0 | 0,0,0 | 0,0,0 | 0,0,0 | 0,0,0 | 0,0,0 | 248/248 (100) |  |
| Screwdriver-shaped | | | 0,0,0 | 0,0,0 | 0,0,0 | 0,0,0 | 0,0,0 | 0,0,0 | 0,0,0 | 0,0,0 | 0,0,0 | 248/248 (100) |  |
| Tuberculum Carabelli | | | 0,0,0 | 0,0,0 | 0,0,0 | 0,0,0 | 0,0,0 | 0,0,0 | 0,0,0 | 0,0,0 | 0,0,0 | 243/248 (98.0) |  |
| Radix relicta | | | 0,0,0 | 0,0,0 | 0,0,0 | 0,0,0 | 0,0,0 | 0,0,0 | 0,0,0 | 0,0,0 | 0,0,0 | 248/248 (100) |  |
| Impaction | | | 0,0,0 | 0,0,0 | 0,0,0 | 0,0,0 | 0,0,0 | 0,0,0 | 0,0,0 | 0,0,0 | 0,0,0 | 248/248 (100) |  |
| Primary retention | | | 0,0,0 | 0,0,0 | 0,0,0 | 0,0,0 | 0,0,0 | 1,1,1 | 0,0,0 | 0,0,0 | 0,0,0 | 248/248 (100) |  |
| Secondary retention | | | 0,0,0 | 0,0,0 | 0,0,0 | 0,0,0 | 0,0,0 | 0,0,0 | 0,0,0 | 0,0,0 | 0,0,0 | 248/248 (100) |  |
|  | | |  |  |  |  |  |  |  |  |  |  |  |
| Orthodontic characteristics | | |  |  |  |  |  |  |  |  |  |  |  |
| Hypodontia | | | 0,0,0 | 0,0,0 | 0,0,0 | 0,0,0 | 0,0,0 | 0,0,0 | 0,0,0 | 0,0,0 | 0,0,0 | 9/9 (100) |  |
| Hyperdontia | | | 0,0,0 | 0,0,0 | 0,0,0 | 0,0,0 | 0,0,0 | 0,0,0 | 0,0,0 | 0,0,0 | 0,0,0 | 9/9 (100) |  |
| Diastema upper | | | 0,0,0 | 0,0,0 | 0,0,0 | 0,0,0 | 0,0,0 | 0,0,0 | 0,0,0 | 0,0,0 | 0,0,0 | 9/9 (100) |  |
| Diastema lower | | | 0,0,0 | 0,0,0 | 0,0,0 | 0,0,0 | 0,0,0 | 0,0,0 | 0,0,0 | 0,0,0 | 0,0,0 | 9/9 (100) |  |
| Crowding lower anterior | | | 1,1,1 | 1,1,1 | 1,1,1 | 0,0,1 | 0,0,0 | 0,0,1 | 0,0,0 | 0,1,1 | 1,1,1 | 6/9 (66.7) |  |
| Crowding upper anterior | | | 0,0,1 | 0,0,0 | 0,0,0 | 0,0,0 | 0,0,0 | 0,0,0 | 0,0,0 | 0,0,0 | 0,0,1 | 7/9 (77.8) |  |
| Deep bite | | | 0,0,0 | 0,0,0 | 0,0,0 | 0,0,0 | 0,0,0 | 0,0,0 | 0,0,0 | 0,0,0 | 0,0,0 | 9/9 (100) |  |
| Anterior open bite | | | 0,0,0 | 0,0,0 | 0,0,0 | 0,0,0 | 0,0,0 | 1,1,1 | 0,0,0 | 0,0,0 | 0,0,0 | 9/9 (100) |  |
| Cross bite | | | 0,0,0 | 0,0,0 | 0,0,0 | 0,0,0 | 0,0,0 | 0,0,0 | 0,0,0 | 0,0,0 | 0,0,0 | 9/9 (100) |  |
|  | Unilateral cross bite | | 0,0,0 | 0,0,0 | 0,0,0 | 0,0,0 | 0,0,0 | 0,0,0 | 0,0,0 | 0,0,0 | 0,0,0 | 9/9 (100) |  |
|  | Bilateral cross bite | | 0,0,0 | 0,0,0 | 0,0,0 | 0,0,0 | 0,0,0 | 0,0,0 | 0,0,0 | 0,0,0 | 0,0,0 | 9/9 (100) |  |
| Molar occlusion | | |  |  |  |  |  |  |  |  |  |  |  |
|  | Class I | | 0,0,1 | 1,1,1 | 1,1,1 | 1,1,1 | 1,1,1 | 1,1,0 | 1,1,1 | 1,1,1 | 1,1,1 | 7/9 (77.8) |  |
|  | Class II | | 0,0,0 | 0,0,0 | 0,0,1 | 1,1,1 | 0,0,0 | 0,0,1 | 0,0,0 | 0,0,0 | 0,0,0 | 7/9 (77.8) |  |
|  | Class III | | 1,1,1 | 0,0,0 | 0,0,1 | 0,0,0 | 0,0,0 | 0,0,0 | 0,0,0 | 1,1,1 | 0,0,0 | 8/9 (88.9) |  |

Figures (x,x,x) are numbers of teeth, which by each of the three examiners were assessed to have the specified characteristics. In section on orthodontic characteristics, the figures are numbers of patients.

*Agreement means the number (n) of teeth (out of 248) or number of patients (out of 9), which were assessed as the same by the three examiners.
